# The peripheral Atf3 ^+^ neuronal population is responsible for nerve regeneration at the early stage of nerve injury revealed by single-cell RNA sequencing: Peripheral *Atf3* ^+^ neuronal population is responsible for nerve regeneration

**DOI:** 10.3724/abbs.2024169

**Published:** 2024-11-13

**Authors:** Li Liu, Junhui Chen, Wen Yin, Po Gao, Yinghui Fan, Daxiang Wen, Yingfu Jiao, Weifeng Yu

**Affiliations:** 1 Department of Anesthesiology Renji Hospital School of Medicine Shanghai Jiao Tong University Shanghai 200127 China; 2 Key Laboratory of Anesthesiology (Shanghai Jiao Tong University) Ministry of Education Shanghai 200127 China

**Keywords:** nerve regeneration, single-cell RNA sequencing, dorsal root ganglion (DRG), nerve injury, *Fgf3*- *Fgfr1* signaling

## Abstract

Peripheral nerve injury (PNI) can transform primary somatosensory neurons to a regenerative state. However, the details of the transcriptomic changes associated with the nerve regeneration of somatosensory neurons remain unclear. In this study, single-cell RNA sequencing (scRNA-seq) is conducted on mouse dorsal root ganglion (DRG) cells after the early stage of nerve injury on day 3 after chronic constriction injury (CCI). We observe that a novel CCI-induced neuronal population (CIP) emerge and express high levels of activating transcription factor ( *Atf3*), a neuronal injury marker. CIP neurons highly express regeneration-associated genes (RAGs) and are enriched in regeneration-related gene ontology (GO) terms, suggesting that these neurons can constitute a pro-regenerative population. Moreover, intercellular communication networks show that CIP neurons closely communicate with satellite glial cells (SGCs) and specifically transmit strong *Fgf3*- *Fgfr1* signaling to SGCs, which could initiate regeneration-associated transcriptional changes in SGCs. We also confirm that regenerative progress occurs at the early stage of nerve injury because immunohistochemistry shows that the expression of ATF3 is significantly increased beginning at 3 days post-CCI and decreased at 1 month post-CCI. Our bioinformatics analysis at single-cell resolution advances the knowledge of regenerative dynamic transcriptional changes in DRG cells after injury and the underlying molecular mechanisms involved.

## Introduction

Peripheral nerve injury (PNI) can result in motor and sensory disorders such as neuropathic pain, which affect 7%–10% of the general population and seriously reduce people’s quality of life [ 1, 2]. Neuronal functional recovery requires axonal regrowth [3]. In contrast to the central nervous system (CNS), the peripheral nervous system (PNS) has intrinsic regeneration and repair abilities [3]. Currently, the preferred treatments are meticulous microsurgical repair by the use of tensionless epineurial sutures and autologous nerve grafting, which requires more extensive procedures and sacrifices the supply of healthy nerves and donor nerves [ 1, 4]. However, although the understanding of neuropathophysiology has greatly improved, the principles of clinical treatment for nerve injury have not changed, and accordingly, clinical outcomes remain poor [5]. Therefore, a deeper understanding of the molecular and cellular mechanisms of nerve regeneration is urgently needed to explore new possibilities for treating nerve injury.

Many molecules and signaling pathways involved in regeneration have been investigated. For example, a previous report indicated that collapsin response mediator protein 4 (CRMP4) plays dual roles in the proximal and distal axon segments of severed DRG neurons to promote axon regeneration [6]. Another study suggested that the Klf2-Vav1-Rac1 axis induced by retrograde Ca ^2+^ signaling from injured axons facilitates axon regeneration through activating Rac1 GTPase in adult DRG neurons [7]. Nevertheless, comprehensive insights into nerve regeneration remain unclear. Single-cell RNA sequencing (scRNA-seq) is a powerful technology for characterizing the transcriptome of individual cells and clarifying biological mechanisms at the cellular level. Recently, scRNA-seq has been used to study transcriptomic perturbations in DRG neurons following nerve injury [ 8, 9]. However, there are still many important questions that have not been adequately addressed, especially the identification of nerve regeneration-associated neuronal subtypes and their mechanisms. CCI is a well-established model that includes compression, ischemia, inflammation, and axonal demyelination [10] and mimics the etiology of clinical conditions and causes symptoms similar to those of posttraumatic neuropathic pain in humans [11].

In the present study, we used a day 3 post-CCI model to study the early stage of nerve regeneration. We characterized transcriptomic perturbations in DRG neurons following chronic constriction injury (CCI) of the sciatic nerve and identified a novel neuron type, the CCI-induced neuronal population (CIP), which is marked by *Atf3*. Compared with other neurons, CIP neurons are characterized by high expression of genes associated with nerve regeneration and repair. Moreover, we studied the gene regulatory networks (GRNs) and communication networks of CIP. Our results may advance the understanding of DRG neuronal subtype-specific treatment for nerve regeneration and provide a basis for the development of nerve regenerative therapy.

## Materials and Methods

### Animals

Naïve male 6–8-week-old C57BL/6J mice obtained from the vivarium of Shanghai Jiaotong University School of Medicine (Shanghai, China) were used in all experiments. The animals weighed 22–26 g and were housed in a temperature-controlled room (22–25°C) illuminated from 07:00 to 19:00. Food and water were available *ad libitum*. The present study was performed in accordance with the Guiding Principles in the Care and Use of Animals and the Animal Management Rule of the Ministry of Public Health, China (documentation 545, 2001) and approved by the Ethics Committee for Experimental Use of Animals of Shanghai Jiaotong University School of Medicine (approval code: #SYXK-2013-0050).

### Surgical procedures for CCI

CCI surgery was performed as described previously [10]. All surgeries were conducted under gaseous anesthesia by using a mixture of 2% isoflurane and oxygen. First, the left thigh region of the mice was neatly shaved and sterilized by using iodine solution. Then, the sciatic nerve was exposed by a blunt incision on the femoral muscle of the biceps of the left paw. The muscle and fascia attached to the sciatic nerve were removed without developing stress on the nerve. The left sciatic nerve was loosely ligated with 4-0 chromic gut sutures in four regions 1 mm apart. The sham procedure involved an equal process without sciatic nerve ligation. Later, the muscle and skin were closed layer by layer using 3-0 silk suture, and iodine solution was applied superficially on the skin.

### Immunohistochemistry

Ipsilateral L4-5 DRGs were harvested 3, 7, 14, 28 days and 2 months after CCI from injured mice and sham mice under anesthesia, immediately fixed with 4% PFA for 6 h and dehydrated with 30% sucrose PBS buffer at 4°C overnight. DRGs were sectioned into 10-μm sections and processed for immunostaining as previously described [12]. The following primary antibodies were used: anti-GFAP (mouse, 1:1000, 3670S; Cell Signaling Technology, Danvers, USA) and anti-ATF3 (rabbit, 1:500, ab254268; Abcam, Cambridge, UK). Sections were then stained with Alexa 594- or Alexa 488-conjugated secondary antibodies (1:1000, ab150080 and ab150105; Abcam) and mounted with DAPI (Thermo Fisher Scientific, Waltham, USA). Slides were imaged on a Slide Scanner microscope (Olympus, Tokyo, Japan).

### Quantitative real-time reverse transcription PCR (qRT-PCR)

qRT-PCR was performed as previously described [13]. The total RNA of the DRG was extracted using Trizol reagent (Invitrogen, Carlsbad, USA) and reverse transcribed with the PrimeScriptTM RT reagent kit with gDNA Eraser (perfect real time) kit (Takara, Dalian, China) following the manufacturer’s instructions. Gene-specific mRNA analyses were performed using the standard protocol of the SYBR premix ex TaqTMII (TliRnaseH plus) kit (Takara). After amplification, each qPCR product was sequenced via electrophoresis to ensure its specificity. The sequences of primers used for RT-PCR were listed in the Supplementary Table S1. The *Gapdh* gene was used as a reference gene to normalize specific gene mRNA expression.

### Tissue dissociation and cell purification

Briefly, L4-5 DRGs from adult mice (8 weeks) were collected in cold complete saline solution (CSS; 137 mM NaCl, 5.3 mM KCl, MgCl _2_ ∙6H _2_O, 25 mM sorbitol, 10 mM HEPES, 3 mM CaCl _2_, pH 7.2) and then incubated in an enzyme solution in CSS composed of 0.35 U/mL Liberase TM (Roche, Basel, Switzerland) and 0.6 mM EDTA TM at 37°C for 20 min, followed by incubation at 37°C for 10 min in CSS composed of 0.35 U/mL Liberase TL (Roche), 0.6 mM EDTA, and 30 U/mL papain (Worthington Biochemical, Lakewood, USA). Enzymatic digestion was stopped by addition of 1 mL DRG media supplemented with 1.5 mg/mL bovine serum albumin (BSA; Sigma, St Louis, USA) and 1.5 mg/mL trypsin inhibitor (Sigma). After gentle trituration with a 1-mL pipette, DRG neurons were centrifuged at 700 *g* for 2–3 min and resuspended in 1 mL DH10 solution (90% DMEM/F-12, 10% FBS, 100 U/mL penicillin, and 100 μg/mL streptomycin; Gibco, Carlsbad, USA). After digestion, the samples were sieved through a 70-μm cell strainer and centrifuged at 300 *g* for 5 min. After the supernatant was removed, the pelleted cells were suspended in red blood cell lysis buffer (Miltenyi Biotec, Bergisch Gladbach, Germany) to lyse the red blood cells. After wash with PBS containing 0.04% BSA, the cell pellets were resuspended in PBS containing 0.04% BSA and refiltered through a 35-μm cell strainer. Dissociated single cells were then stained with AO/PI for viability assessment using a Countstar Fluorescence Cell Analyzer (Inno-Alliance Biotech, San Diego, USA).

### Single-cell sequencing

The scRNA-seq libraries were generated using the 10× Genomics Chromium Controller Instrument and Chromium Single Cell 3’ V3.1 Reagent kits (10× Genomics, Pleasanton, USA). Briefly, cells were concentrated to approximately 1000 cells/μL and loaded into each channel to generate single-cell Gel Bead-In-Emulsions (GEMs). After the reverse-transcription step, the GEMs were broken, and barcoded cDNA was purified and amplified. The amplified barcoded cDNA was fragmented, A-tailed, ligated with adaptors and subjected to index PCR amplification. The final libraries were quantified using the Qubit High Sensitivity DNA assay kit (Thermo Fisher Scientific), and the size distribution of the libraries was determined using a high sensitivity DNA chip on a Bioanalyzer 2200 (Agilent, Santa Clara, USA). All libraries were sequenced on an Illumina sequencer (Illumina, San Diego, USA) on a 150 bp paired-end run.

### Single-cell RNA statistical analysis

scRNA-seq data analysis was performed by NovelBio Co., Ltd. with the NovelBrain Cloud Analysis Platform ( www.novelbrain.com). We applied fastp with default parameter filtering of the adaptor sequence and removed the low-quality reads to obtain clean data. Then, the feature-barcode matrices were obtained by aligning reads to the mouse (mm10, Ensembl100) using CellRanger v5.0.1. We applied downsample analysis to the samples sequenced according to the mapped barcoded reads per cell of each sample and finally obtained the aggregated matrix. When more than 500 genes were expressed and the mitochondrial UMI percentage was less than 20%, the cells were subjected to cell quality filtering, and mitochondrial genes were removed from the expression table.

The Seurat package (version: 3.1.4, https://satijalab.org/seurat/) was used for normalization of the scRNA-seq data. Cell normalization and regression were conducted based on the expression table using the SCTransform function, considering the UMI counts of each sample and percentage of mitochondrial reads to obtain the scaled data. The SelectIntegrationFeatures, PrepSCTIntegration, and FindIntegrationAnchors functions were then applied to identify highly variable features across cells and to establish “anchors” for integrating individual datasets. This integration process resulted in the creation of an “unbatched” dataset through the IntegrateData function, effectively removing batch effects and enabling combined analysis of all cells.

PCA was constructed based on the scaled data with the top 2000 highly variable genes, and the top 10 principal components were used for tSNE construction and UMAP construction. We identified DRG cell type clusters by initially employing a graph-based clustering method (resolution = 0.8) to obtain cell clustering results, as detailed in Supplementary Figure S1A. Marker genes were then determined using the FindAllMarkers function with the Wilcoxon rank-sum test algorithm under the following criteria: i) lnFC > 0.25, ii) *P* value < 0.05, and iii) min.pct > 0.1. If adjacent clusters exhibited similar expression of specific marker genes, we merged them, as illustrated in Supplementary Figure S1B. Subsequently, we categorized the cell clusters into different types based on marker gene expression, as depicted in Supplementary Figure S2A, thereby identifying the DRG cell types.

Similarly, for DRG neuronal subtype identification, we utilized a graph-based clustering method (resolution = 0.8) to generate neuronal clustering results, as outlined in Supplementary Figure S3A. Marker genes were computed via the FindAllMarkers function employing the Wilcoxon rank-sum test algorithm. If adjacent clusters showed analogous expression of specific marker genes, we consolidated them. In cases where one cluster exhibited heterogeneity with varying markers across cells, we subdivided it and classified the resulting subclusters into different types, as demonstrated in Supplementary Figure S3B. We subsequently categorized the neuronal subclusters based on marker gene expression to identify the DRG neuronal subtypes.

### Cell communication analysis

CellChat analysis was performed using the R package CellChat. First, we used the function ‘createCellChat’ to create a CellChat object for the overall gene expression matrix, and then we used CellChatDB.mouse provided by CellChat as the mouse ligand receptor reference. Finally, the cell-cell communication probability was inferred using the function ‘computeCommunProb’, and the communication probability at the level of each cell signaling pathway was inferred by the function ‘computeCommunProbPathway’.

### SCENIC analysis

To assess transcription factor regulation strength, we applied the single-cell regulatory network inference and clustering (pySCENIC, v0.9.5) workflow using the 20-thousand motif database for RcisTarget and GRNboost.

### Differential gene expression analysis

To identify differentially expressed genes (DEGs) among the samples, the function FindMarkers with the Wilcoxon rank sum test algorithm was used under the following criteria: i) lnFC > 0.25, ii) *P* value < 0.05 and iii) min.pct > 0.1.

## Results

### scRNA-seq sequencing of mouse DRGs

**Figure 1:**
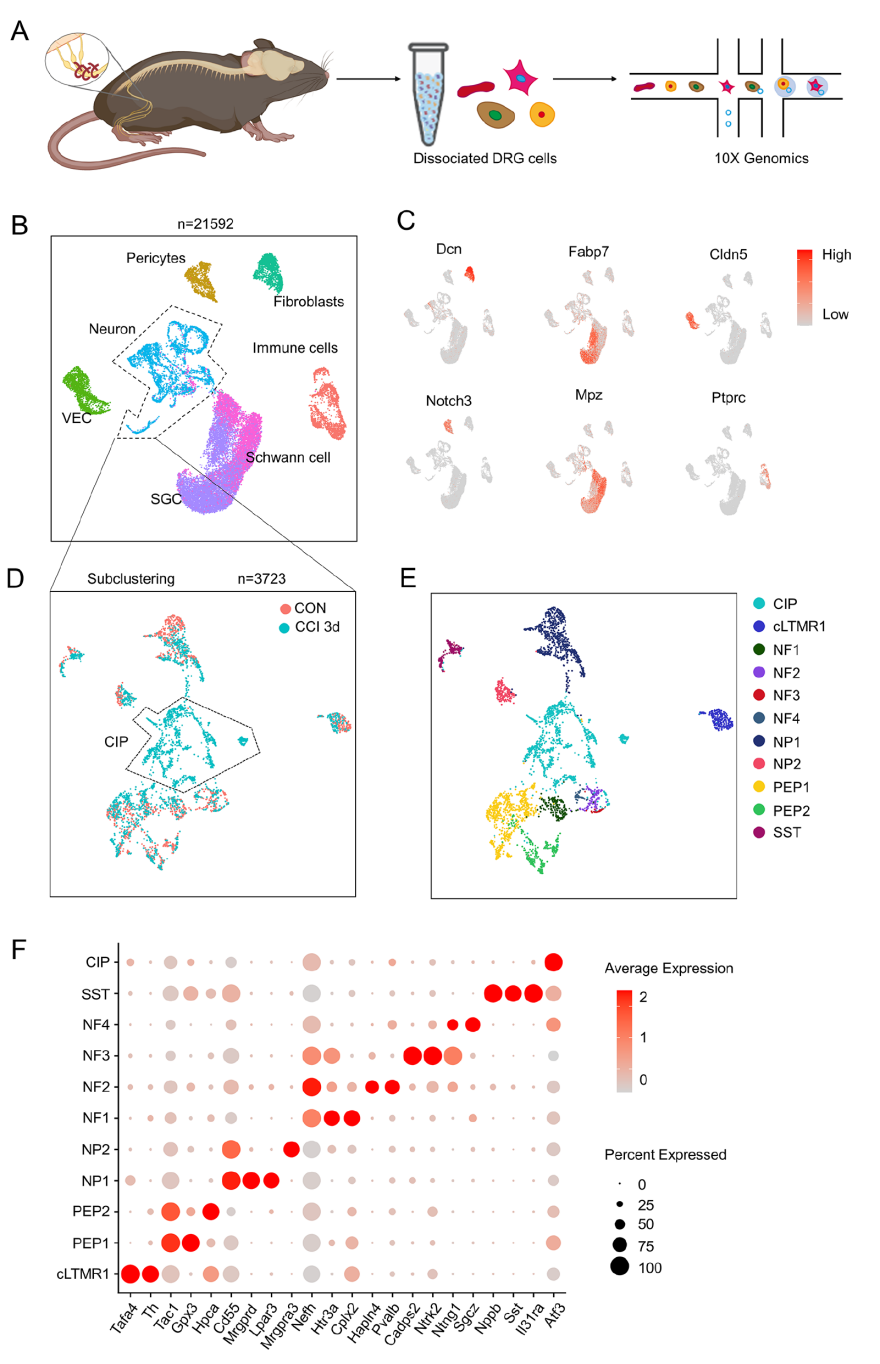
Figure 1 Seven types of DRG cells were identified based on their expression of marker genes (A) Experimental design. L4 and L5 DRGs were collected from CON (naïve state) mice and CCI 3d mice, and the dissociated cell suspension was processed via 10× Genomics scRNA-seq. (B) UMAP plot of DRG cells profiled in the present study. Each dot represents an individual cell; each cell type is marked by a unique color. (C) Feature heatmap showing the expression patterns of cell type-specific marker genes in all cell types. The color represents the expression level. (D) UMAP plot showing neuronal subpopulations in the CON and CCI groups. Pink dots represent neurons in the CON group, and turquoise dots represent neurons in the CCI group. (E) UMAP plot of neuronal subpopulations. Each color represents a neuronal subtype. (F) Bubble plot of marker genes of 11 neuronal subpopulations. The color represents the average expression level of the marker genes, and the size represents the percentage of neurons expressing the marker genes.

We conducted scRNA-seq on cells dissociated from the left lumbar (L) 4–5 DRGs of control (CON) mice and CCI mice ( Figure 1A). CCI-operated mice exhibited a significant decrease in the paw withdrawal threshold ( Supplementary Figure S2B). After quality control, a total of 21,592 cells were obtained from 4 samples, including from CCI mice and CON mice. Based on gene expression patterns, we mapped all cells into seven major cell types with distinct markers and classified them by uniform manifold approximation and projection (UMAP) ( Figure 1B). These cells include neurons ( *Rbfox3* ^+^), satellite glial cells ( *Fabp7 ^+^*), fibroblasts ( *Dcn ^+^*), pericytes ( *Notch3 ^+^*), immune cells ( *Ptprc ^+^*), schwann cells ( *Mpz ^+^*), and endothelial cells ( *Cldn5 ^+^*) ( Figure 1C). This classification is consistent with previous reports [14] .

To explore the heterogeneity of DRG neurons under normal and CCI neuropathic conditions, we reclassified 3723 neurons in the 2 groups based on canonical DRG neuron markers [15]. Eleven neuronal subtypes were visualized in UMAP, and their DEGs were identified by the Seurat package ( Figure 1D,E and Supplementary Figure S3B). These neurons included *Tac1 ^+^*/ *Gpx3 ^+^* peptidergic neurons (PEP1), *Tac ^+^* / *Hpca ^+^* peptidergic neurons (PEP2), *Cd55 ^+^*/ *Mrgprd ^+^* non-peptidergic neurons (NP1), *Cd55 ^+^*/ *Mrgpra3 ^+^* non-peptidergic neurons (NP2), *Nefh ^+^* neurons (NF1, NF2, NF3, and NF4), and *Sst ^+^*/ *Nppb ^+^* pruriceptors (SSTs). In addition, we identified a novel CCI-induced neuronal population in the CCI group. CIP was marked by *Atf3* ( Figure 1D–F), which was induced following nerve injury and is functionally associated with nerve regeneration [ 16, 17]. The classification of neuronal subtypes was in line with previous scRNA-Seq studies [15].

### Changes in the transcriptome characteristics of neurons after CCI

**Figure 2:**
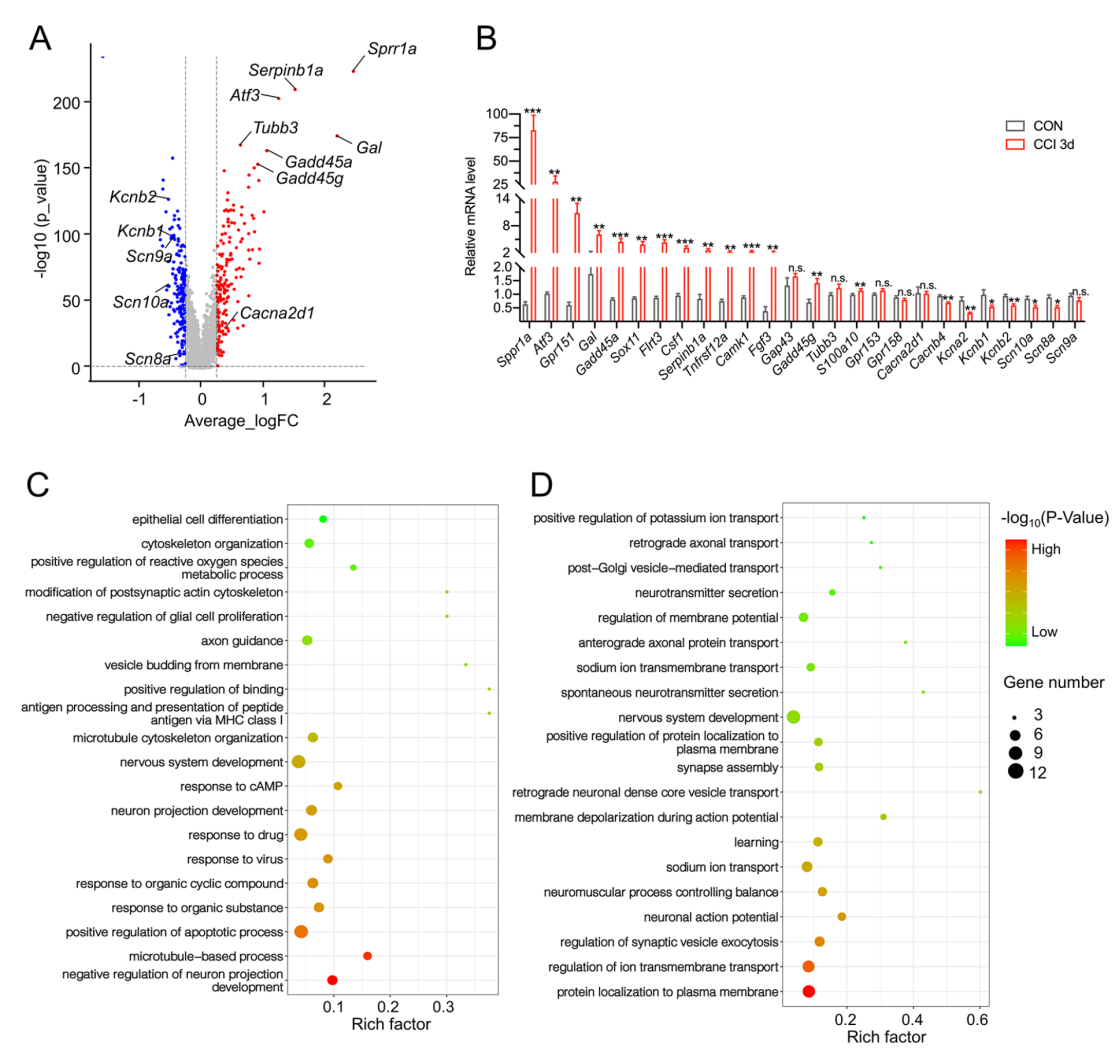
Figure 2 Transcriptional reprogramming of DRG neurons is related to nerve regeneration (A) Volcano dot plot of DEGs in the DRGs of the CON and CCI 3d groups. The red dots represent upregulated genes under CCI, the blue dots represent downregulated genes under CCI, and the gray dots represent genes whose expression was not significantly different between CON and CCI 3d mice. Each dot represents a gene. (B) Some significantly differentially expressed genes were confirmed via RT-PCR. Each group comprised 5 samples. *P < 0.05; **P < 0.01; ***P < 0.001; unpaired t test. Data are presented as the mean ± SEM. (C) Bar plot showing the top 20 GO terms of biological process (BP) enriched for the upregulated DEGs in CCI 3d. (D) The top 20 BP-enriched GO terms for the downregulated DEGs in the CCI 3d group.

To characterize the common transcriptomic changes in neurons following CCI-induced axonal injury, a volcano plot was generated to visualize the DEGs of neurons. We observed that CCI greatly upregulated the expressions of *Sprr1a*, *Atf3*, *Gap43*, *Flrt3* , *Gal*, *Sox11*, *Gadd45a*, *Gpr151*, and *Serpinb1a* ( Figure 2A), which were identified as regeneration-associated genes (RAGs). These RAGs play a decisive role in the regeneration of damaged axons [18]. In addition, CCI downregulated multiple potassium and sodium channels and upregulated the calcium channel Cacna2d1, consistent with previous studies in nerve transection models [9]. These gene changes could affect neuronal excitability and result in ectopic discharge after nerve injury [19]. qRT-PCR was utilized to validate the changes in the DEGs ( Figure 2B). PCR results showed that all of the aforementioned RAGs significantly increased, while potassium and sodium channels significantly decreased ( Figure 2B). *Sprr1a* was one of the top DEGs, increasing more than 100-fold at CCI 3d. A previous study reported that *Cacna2d1* expression decreased on day 28 post-CCI [20], but this study showed that *Cacna2d1* expression did not significantly differ at CCI 3d ( Figure 2B). GO analysis revealed that the DEGs upregulated in neurons after CCI were enriched mainly in regeneration-related terms, such as nervous system development, microtubule cytoskeleton organization, and neuron projection organization ( Figure 2C). In addition, the downregulated DEGs were significantly related to peripheral stimuli and signal transduction, including protein localization to the plasma membrane, ion transmembrane transport, and synaptic vesicle exocytosis ( Figure 2D). These downregulated genes included the ion channel genes *Kcnb2*, *Kcnb1*, *Scn9a*, *Scn10a*, *Scn8a* and *Pacsin1*, which are important regulators of the actin cytoskeleton and endocytosis and are highly expressed in neurons [ 21, 22].

### Transcriptomic characteristics of the novel CIP neurons

**Figure 3:**
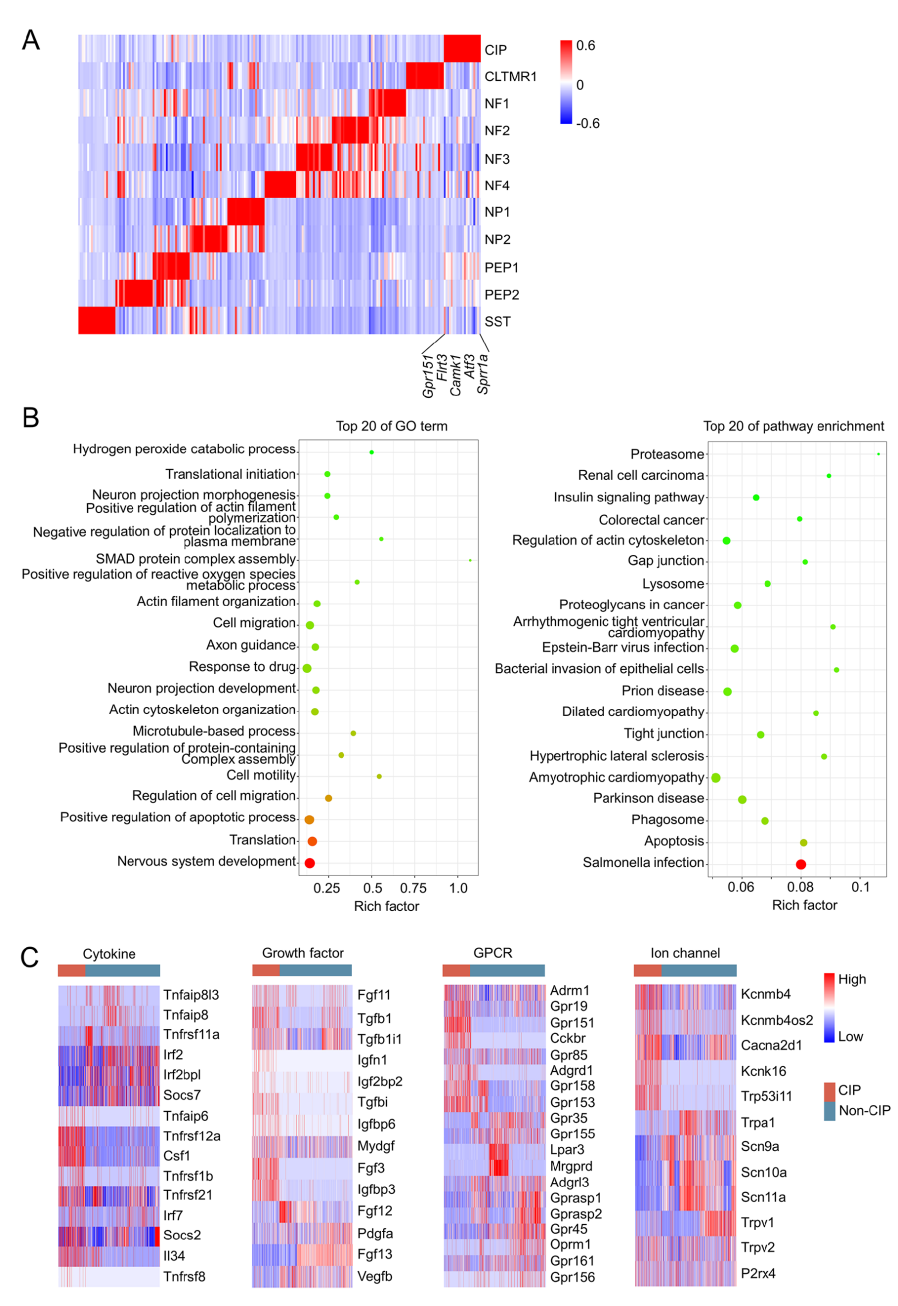
Figure 3 A novel CCI-induced neuronal subpopulation is associated with nerve regrowth (A) Heatmap showing the DEGs among 11 neuronal subpopulations. The color represents the average expression level of the marker genes. (B) Bubble plots showing the top 12 GO terms of BP and the top 12 pathway enrichment terms enriched for the DEGs of CIP neurons. (C) Heatmaps of 4 DEG modules of CIP neurons versus Non-CIP neurons.

The UMAP plot was split according to the CON and CCI groups to investigate the development and progression of the novel emerging neuron type CIP ( Figure 1D,E). Other neuronal subpopulations were evenly distributed ( Figure 1D,E). DEGs of 11 neuronal subpopulations were identified (lnFC>0.25 and *P* value<0.05). ( Figure 3A). CIP was represented by *Sprr1a*, *Atf3*, *Camk1* , *Flrt3* and *Gpr151* ( Figure 3A). These genes were well-known RAGs and were reported to be upregulated in previous studies [ 23, 24]. We conducted GO analysis to study the functions of the DEGs associated with CIP. Here, we showed the top 20 GO terms ( Figure 3B). These representative genes in the CIP were significantly enriched in nerve regeneration-related terms (nervous system development, actin cytoskeleton organization, neuron projection development, axon guidance, actin filament organization, neuron projection morphogenesis, etc.), increased translation, and apoptotic processes ( Figure 3B). However, the top terms in the CIP category excluded biological processes associated with pain. Furthermore, the top 20 pathways enriched in CIP were mainly involved in apoptotic signaling and gap junction signaling ( Figure 3B).

To characterize the transcriptomic pattern of CIP molecules, the DEGs of CIP neurons were divided into four groups: genes related to cytokines and their related receptors, genes related to growth factors and their related receptors, G protein-coupled receptors (GPCRs), and genes related to ion channels. We observed that CIP exhibited unique transcriptome pattern features ( Figure 3C). Real-time PCR showed that *Tnfrsf12a*, *Csf1* and *Fgf3* were upregulated in the DRG after CCI ( Figure 2B). Herein, scRNA-seq showed that these genes were specifically expressed in CIP. In addition, in the cytokine and growth factor module, *Tnfrsf8*, *Igfn1*, *Tgfbi*, and *Igfbp3* were also specifically expressed in CIP. In the GPCR module, *Gpr151* and *Cckbr* are highly specifically expressed in CIP. These CIP-specific genes are enriched in nerve regeneration and have been shown to play roles in axonal growth [ 25, 26 ]. *Tgfbi* and *Igfbp3* play significant roles in embryonic development, cell proliferation and differentiation, adhesion, migration, *etc*. [ 27, 28]. Increased *Fgf3* could play a protective role in suppressing the development of neuronal hyperexcitability after CCI [29]. *Gpr151* was reported to be engaged in neuropathic pain [30]. Our real-time PCR showed that ion channel genes ( *e*. *g*., sodium and potassium channels) were widely downregulated following CCI, as previously reported ( Figure 2B) [ 14, 29]. Specifically, CIP exhibited increased potassium channel expression ( *Kcnmb4*, *Kcnmb4os2*, and *Kcnk16*) and decreased sodium channel expression ( *Scn9a*, *Scn10a*, and *Scn11a*) ( Figure 3C). In addition, *Trpv1* and *Trpa1* (nonselective cation channels related to neuronal hyperexcitability and pain transduction) also exhibited decreased expression in the CIP ( Figure 3C). These genes largely affect neuronal excitability and are related to pain transduction.

### Pivotal regulons and gene regulatory networks in CIP

**Figure 4:**
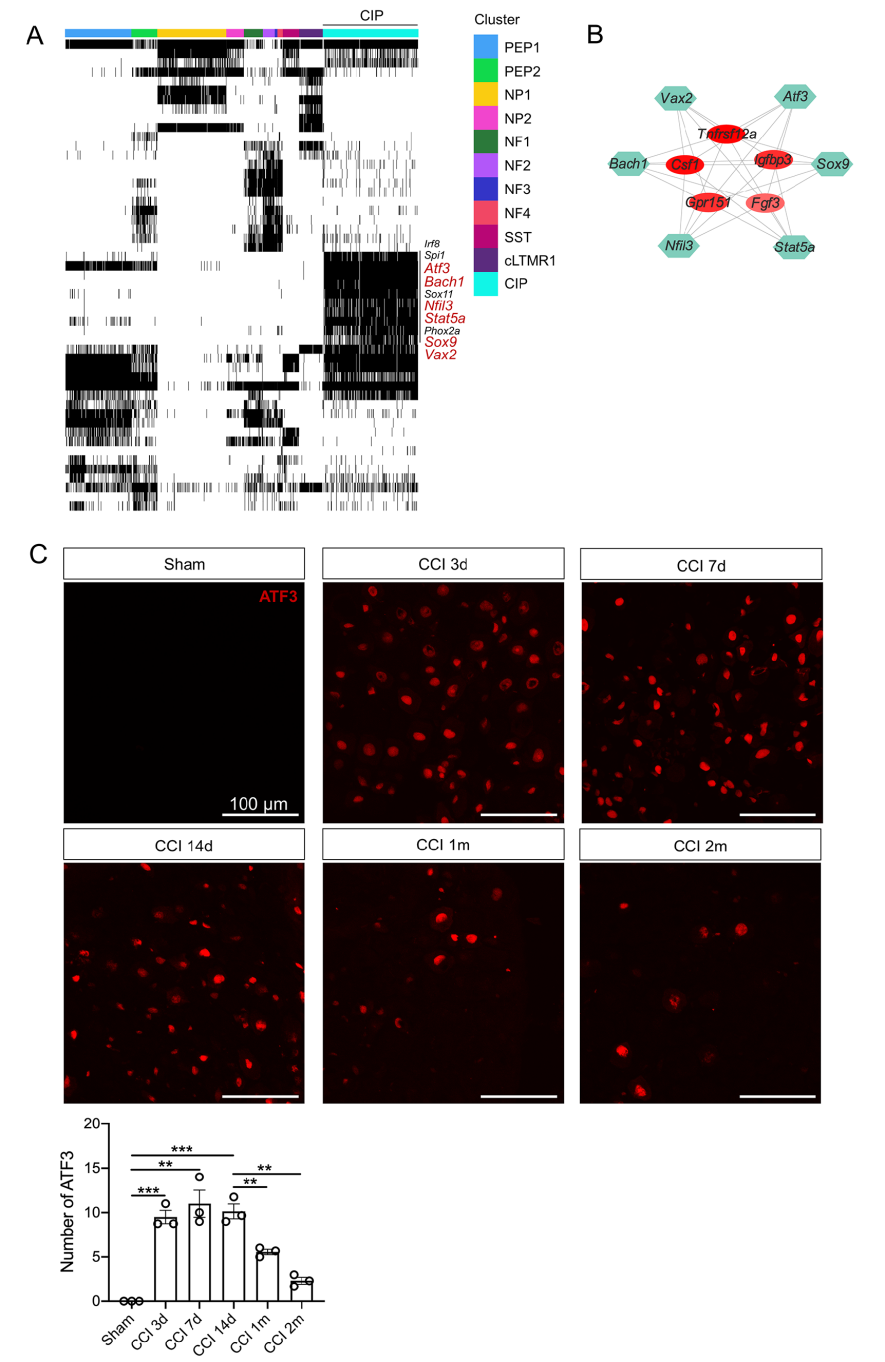
Figure 4 The regulons and GRNs of CIP neurons are related to nerve regeneration (A) The binary heatmap showing the activity of inferred TFs across neuronal subpopulations. (B) GRNs of the CIP-specific regulons Atf3, Bach1, Nfil3, Stat5a, Sox9 and Vax2. (C) Immunofluorescence images of L4–L5 DRGs from sham mice and CCI mice at different injury time points (3 mice per group) stained with an anti-ATF3 antibody. Scale bar: 100 μm. Data are shown as the mean ± SEM. ** P < 0.01, ***P < 0.001.

SCENIC analysis was applied to identify the key regulons and construct GRNs of CIP. The binary regulon activity matrix revealed that 6 TFs, namely, *Atf3*, *Bach1*, *Nfil3*, *Stat5a* , *Sox9* and *Vax2*, were CIP-specific regulons ( Figure 4A). Other TFs, including *Irf8*, *Spi1*, *Sox11*, *Phox2a*, and *Sox9*, were also positively activated in CIP. However, *Spi1* was also strongly activated in PEP1/PEP2. *Irf8*, *Sox11* and *Phox2a* were slightly activated in PEP1/PEP2, and *Sox9* was positively activated in NF4 ( Figure 4A). We constructed GRNs for 6 CIP-specific regulons and observed that these TFs collectively regulated the genes *Csf1*, *Tnfrsf12a*, *Igfbp3*, *Gpr151*, and *Fgf3* ( Figure 4B).

Next, we performed immunofluorescence staining for ATF3 in DRGs from sham mice and CCI mice at 3 d, 7 d, 14 d, 1 month, and 2 months. Immunohistochemistry suggested that ATF3 expression significantly increased as early as 3 d after CCI and gradually decreased beginning 1 month after CCI ( Figure 4C). A previous study reported that in rodent models of nerve regeneration, the capacity of chronically denervated distal stumps to support axonal outgrowth is compromised by 8 weeks post-injury and becomes virtually non-existent by 6 months [31]. The time course of the changes in ATF3 expression paralleled the capacity of denervated distal stumps to support axonal outgrowth. These results indicated that CIP neurons may play a key role in nerve regeneration post-CCI.

### Inference and visualization of intercellular communications of CIP

**Figure 5:**
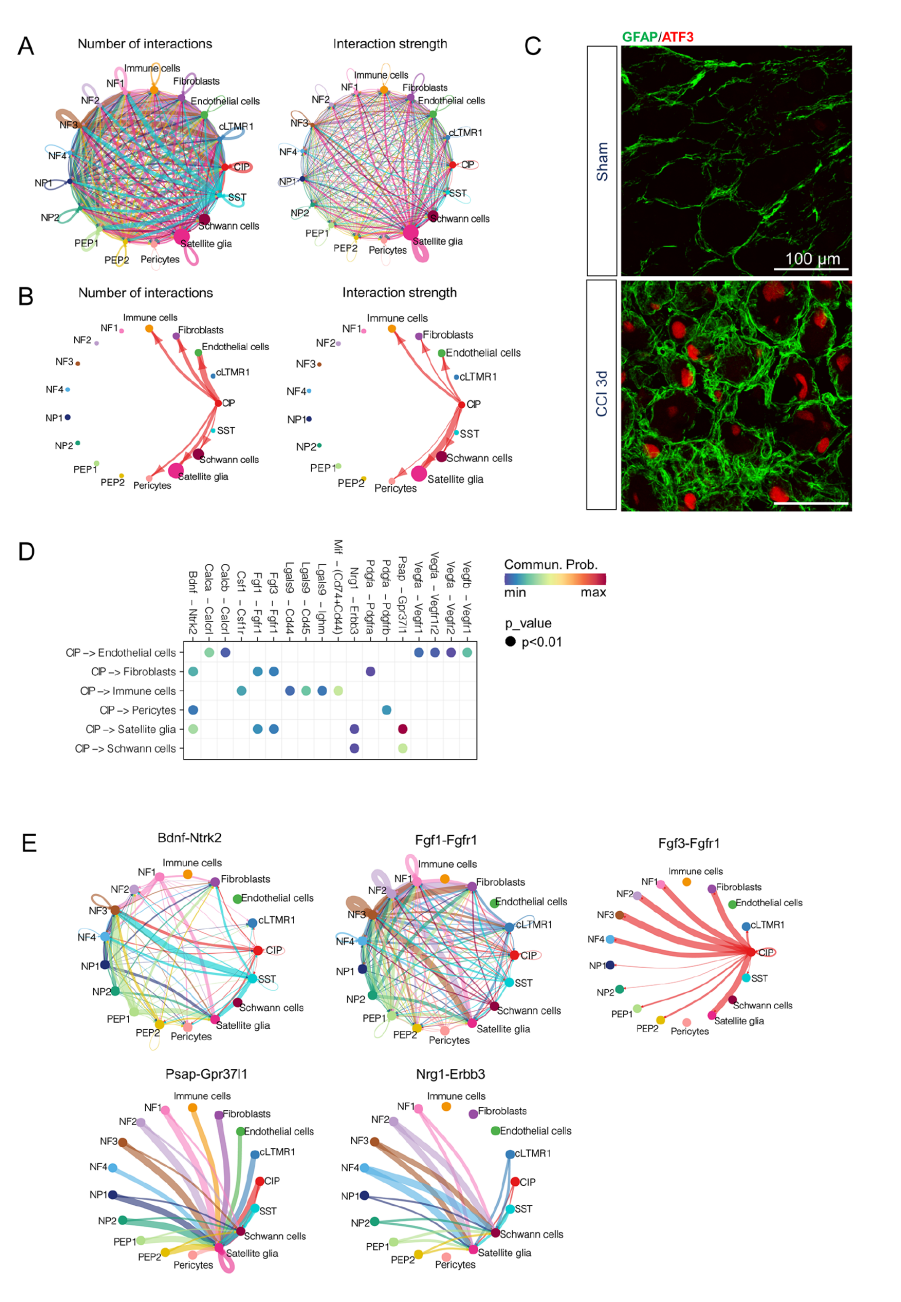
Figure 5 Intercellular communication between CIP and other cell types (A) Number/strength of intercellular communication networks among clusters. Nodes, cell clusters; node size, cell counts; edge width, numbers/strength of interaction. (B) Intercellular communication network showing the number/strength of interactions between CIP and nonneuronal clusters. (C) Immunofluorescence images of L4–L5 DRGs from sham mice and CCI mice stained with antibodies against ATF3 and GFAP. Scale bar: 100 μm. (D) Dot plot showing the probabilities of ligand-receptor pair interactions with significant changes (P < 0.01) when CIPs are sender cells. Commun. Prob, communication probability. The communication probability here equals the interaction strength. The dot color represents the communication probability, and the dot size represents the computed P value. (E) Representative cell-cell communications transmitted from CIP.

CellChat was used to identify significant communications of CIP. The circle plot provided an overview of the inferred intercellular communication network between cell groups ( Figure 5A). According to the interaction number plot of CIP, CIP was closely linked to endothelial cells, SGCs, and fibroblasts. Furthermore, in the interaction strength plot of CIP, CIP was connected to SGCs, immune cells, endothelial cells, *etc*., while the interaction strength of CIP-SGCs was significantly greater than that of other neuronal subtypes, suggesting a strong connection between CIP and SGCs ( Figure 5B).

SGCs have been highlighted for their significant roles in the process of axonal regeneration following nerve injury in previous studies [ 32, 33]. GFAP is a marker of SGC activation [34]. Immunohistochemical staining for GFAP revealed significantly greater activation of SGCs in the DRGs of mice 3 days post-CCI than in those of sham-operated mice, while activated SGCs tightly encased ATF3 ^+^ neurons ( Figure 5C). However, how distant axon injury activates SGCs in the DRG and promotes transcriptomic changes to facilitate axonal regeneration in SGCs is still unclear. DRG neuronal somas can communicate with SGCs through ligand-receptor connections induced by soma-released transmitters [35]. GO analysis also suggested that SGCs exhibited an increased response to extracellular biological information ( Supplementary Figure S4). To identify the specific ligands or receptors involved in this communication, the L-R pairs between CIP and other cells were examined when CIP served as the signaling source. The L-R pairs with the most pronounced changes ( *P* value<0.01) were displayed ( Figure 5D). The analysis indicated that CIP may communicate with SGC via several L-R pairs, including *Psap*- *Grp37l1*, *Bdnf*- *Ntrk2*, *Fgf3*- *Fgfr1* , *Fgf1*- *Fgfr1*, and *Nrg1-Erbb3* ( Figure 5D). Furthermore, by examining the interconnections of these five signaling pathways among all cell types, we observed that only *Fgf3-Fgfr1* was specifically sent from CIP neurons and was primarily transmitted to SGCs ( Figure 5E).

## Discussion

Chronic constriction of the peripheral nerve is a common type of nerve injury that triggers a series of cellular and molecular responses, leading to the conversion of mature sensory neurons into actively developing cells [16]. Nerve regeneration-related events play a significant and predominant role in transcriptional reprogramming [9]. In this study, we utilized 10× scRNA-seq to generate a comprehensive cell atlas of DRGs from CON and CCI 3d mice, aiming to investigate the transcriptomic changes in neurons and gain comprehensive mechanistic insights into the cell type-specific progress of nerve regeneration. Herein, we identified a group of novel nerve regeneration-related CIP neurons and clarified their special transcriptomic characteristics. Additionally, the GRNs of the CIP were identified. Finally, we used the CellChat database to construct intercellular communication networks of CIP and suggested that *Fgf3-Fgfr1* signaling is specifically sent from CIP neurons and strongly transmitted to SGCs.

Due to the regenerative characteristics of peripheral neurons, extensive sequencing studies have been conducted on DRG neurons in the past to generate deeper insights into the reinnervation process. Bulk RNA-seq of DRG tissues revealed significant transcriptomic changes following nerve injury [ 16, 36, 37]. However, transcriptomic reprogramming that occurs in specific cell subtypes can not be discerned because dissociated DRG tissue comprises a mixture of different neuronal subtypes and nonneuronal cells. Recently, through the utilization of snRNA-seq, Renthal *et al*. [9] revealed the neuronal transcriptional program that is activated in response to axonal regeneration under peripheral axotomy. However, snRNA-seq exclusively captures RNA within the nucleus, resulting in a loss of transcriptomic information in the cytoplasm and the use of cell dissociation procedures that themselves induce injury-like and immediate early gene responses. Furthermore, scRNA-seq was recently also conducted to study specific alterations in gene expression within distinct DRG cell subtypes after nerve injury [ 14, 29], revealing subtype-specific perturbations in gene expression in the DRG following nerve injury. Nevertheless, these studies overlooked the crucial role that nonneuronal cells play in axon regeneration. The regeneration of axons in peripheral nerves is not cell autonomous, and the regenerative capacity of peripheral somatosensory neurons relies on the extracellular environment [38]. In this study, we used the CCI as a neuropathic pain model to not only identify a reinnervation-associated CIP neuronal subtype and its inferred gene regulatory networks but also construct cellular communication networks of the CIP and revealed its receptor‒ligand pairs for transmitting signals to other cells.

Our study suggested that bulk transcriptomic changes in mouse DRG neurons upon CCI were similar to previous sequencing reports for neurons from DRGs following various kinds of nerve injury, such as spared nerve injury (SNI) [14], spinal nerve transection, sciatic nerve transection+ligation (ScNT) and sciatic crush [9]; downregulated genes related to neuronal activity, such as those encoding ion channels ( *e*. *g*., *Scn9a*, *Scn10a*, *Scn11a*, *Kcna2*, *Kcnb1* and *Kcnb2*); and downregulated genes involved in neuronal maintenance; upregulated TFs ( *e*. *g*., *Atf3*, and *Sprr1a*) and proteins ( *e*. *g*., *Fgf3*, *Gpr151*, *Flrt3*, and *Gap43*) associated with growth promotion [16] ( Figure 2A). The structure and function of voltage-gated sodium/potassium channels play a critical role in pain transmission [ 39, 40]. For example, a gain of function in the mutations Na _v_1.7, Na _v_1.8, and Na _v_1.9 (encoded by *Scn9n*, *Scn10n*, and *Scn11n*, respectively) renders DRG neurons hyperexcitable and causes peripheral neuropathic pain [ 41– 43]. In addition, dysfunction of potassium channels is related to a hyperexcitable phenotype [44]. GO analysis indicated that the DEGs upregulated in neurons after CCI were enriched mainly for regeneration-related terms, *e.g*., nervous system development, microtubule cytoskeleton organization, and neuron projection organization ( Figure 2C). The downregulated DEGs were significantly related to peripheral stimulus and signal transduction, including protein localization to the plasma membrane, ion transmembrane transport, and synaptic vesicle exocytosis ( Figure 2D). Furthermore, we validated the expressions of DEGs in neurons after CCI by RT-PCR, based on the scRNA-seq data ( Figure 2B). The RT-PCR results were consistent with the results of the scRNA-seq analysis. However, *Cacna2d1* exhibited significantly increased expression in the scRNA-seq data under CCI, with no significant change in the PCR results ( Figure 2 A,B). *Cacna2d1* encodes the voltage-dependent calcium channel subunit alpha-2/delta-1 (α2δ-1). A previous study reported that DRG neurons exhibit an increase in the expression level of α2δ-1 after peripheral axotomy and play a role in neuropathic pain [45]. Further exploration is needed to investigate the unique gene expression patterns among different nerve injuries, which will contribute to the study of distinct molecular mechanisms involved in nerve regeneration.

Our analysis identified 7 cell types ( Figure 1B) and classified neurons into 11 subtypes ( Figure 1E) according to the expression of canonical DRG markers ( Figure 1C,F) [15]. Eleven neuronal subtypes were evenly distributed according to the UMAP plot ( Figure 1D,E). In this study, we observed a novel regeneration-associated neuron type following CCI, namely, CIP ( *Atf3* ^+^) neurons, and identified their transcriptomic characteristics and intercellular communication. *Atf3*, a TF that was demonstrated to be necessary for nerve injury-induced transcriptional reprogramming, axonal regeneration and sensory recovery following injury [ 46, 47], was significantly induced following CCI ( Figure 4C). In addition, the top 20 DEGs of CIP included RAGs that are important for nerve regeneration, such as *Gpr151* , *Flrt3*, *Gadd45a*, and *Spprr1a* ( Figure 3A). In addition, we observed that some functional genes related to nerve regeneration were specifically expressed in the CIP ( Figure 3C). The expression of *Tnfrsf12a*, a weak tumor necrosis-like inducer of apoptosis receptors, was upregulated after peripheral nerve damage and promoted neurite outgrowth [25]. *Csf1* was enriched in the inflammatory response associated with pathological pain. A previous study reported that *Csf1* supported axonal revival and myelin sheath reconstruction after injury to the central nervous system (CNS) [48]. Moreover, whether *Csf1* plays a role in nerve regeneration after peripheral nerve injury is controversial, and further research is needed [49]. Moreover, *Gpr151* expression is strongly increased after nerve injury and is known to upregulate the expression of *Csf1* [50]. The fasciclin family extracellular matrix protein *Tgfbi* is required for embryogenesis [51]. *Igfbp3*, an insulin-like growth factor (IGF)-binding protein 3, enhances the survival of neurons by regulating the effects of IGFs [ 52, 53]. Interestingly, CIP was associated with increased levels of potassium channels ( *Kcnmb4* and *Kcnk16*) and decreased levels of sodium channels ( *Scn9a*, *Scn10a*, and *Scn11a*), which could protect CIP-self from hyperexcitability. This may be necessary for transcribing genes essential for nerve regeneration at a sufficient scale.

The enrichment analysis suggested that the top 20 biological processes significantly enriched in the CIP were involved in nerve regeneration, including “nervous system development”, “actin cytoskeleton organization”, “neuron projection development”, “axon guidance”, “actin filament organization”, and “neuron projection morphogenesis”. Furthermore, we conducted pathway enrichment analysis of CIP to explore the mechanisms of regrowth. The top 20 terms included “regulation of actin cytoskeleton” and “gap junction”. The actin cytoskeleton has been reported to play a key role in promoting axonal regrowth [54]. Additionally, the number of gap junctions between neurons and nonneuronal cells increases following axonal injury [ 55, 56], suggesting that intercellular communication between CIP and other cells may contribute to nerve regeneration.

According to the binary heatmap of regulon activity, we discovered that 6 TFs ( *Atf3*, *Bach1*, *Nfil3*, *Stat5a* , *Sox9* and *Vax2*) exhibited specific activation in CIP and regulated the significant DEGs of CIP, including *Fgf*3, *Csf1*, *Tnfrsf12a*, *Igfbp3*, and *Gpr151*. In addition, the temporal pattern of changes in ATF3 expression was closely correlated with axonal outgrowth, which may also suggest that CIP plays a critical role in nerve regeneration.

Next, we conducted cellular communication analysis of CIP to gain valuable insights into the mechanism of nerve regeneration in CIP and found that there was a close link between CIP and SGCs in comparison to other nonneuronal cells. In the DRG, SGCs promote nerve regeneration through multiple mechanisms after injury. For example, nerve injury-induced peroxisome proliferator-activated receptor (PPARα) signaling downstream of fatty acid synthase (FASN) in SGCs contributes to orchestrating nerve regeneration [33]. In addition, vimentin activity in SGCs determines the expression of neural intermediate filaments (IFs) that participate in axonal regeneration in DRG neurons following axon injury [57]. GO analysis also indicated that SGCs were enriched in genes related to “regulation of cell population proliferation”, “axon regeneration” and “cellular response to fibroblast growth factor stimulus” under CCI ( Supplementary Figure S4). However, how nerve injuries at distant axons activate the nerve regeneration-associated transcriptome program of SGCs in the DRG remains largely unknown. Neuronal somas in the DRG can communicate with SGCs through the activation of receptors induced by soma-released transmitters [35]. GO analysis indicated that CIP was involved in gap junction signaling ( Figure 3B). In addition, ATF3 ^+^ neurons were shown to be encased by activated SGCs ( Figure 5C). Hence, we hypothesize that CIP may activate SGCs to promote nerve regeneration.

We found that CIP can send signaling to SGCs mainly *via* *Psap* - *Grp37l1* and *Fgf3*- *Fgfr1*. *Psap*- *Grp37l1* was the strongest signaling pathway from CIP to SGCs and was also involved in the interaction between other neuronal subtypes and SGCs. *Fgf3*- *Fgfr1* was shown to be specifically transmitted from CIP. Hence, we hypothesize that CIP may specifically activate SGCs *via* *Fgf3*- *Fgfr1* to facilitate axonal regrowth.

However, there were some limitations in the present study. The methodological approaches used in our study were not comprehensive enough. Further cellular experiments and animal models are needed to confirm the role of CIP neurons in promoting nerve regeneration postinjury. Additionally, we need to validate whether CIP neurons can influence the activation of SGCs through the *Fgf3*- *Fgfr1* pathway. We aimed to identify novel targets for promoting nerve regeneration through further cellular and animal experiments.

In summary, this study elucidated the single-cell transcriptomic changes in somatosensory neurons following peripheral nerve injury, identified a novel type of CIP in neurons, and investigated the transcriptomics, regulons and cellular communication of this novel type of neuron. In addition, *Fgf3*- *Fgfr1* signaling was inferred to be the specific pathway transmitted by CIP to facilitate pro-regeneration-associated transcriptional changes in SGCs. Thus, this study advances the current understanding of peripheral nerve regeneration, which can be a resource for developing regenerative therapies.

## Supporting information

24273Supplementary_figure_legends-20241029

Supplementary_Table_S1

## Supplementary Data

Supplementary Data is available at *Acta Biochimica et Biophysica Sinica* online.
