# Supplementary material for: The peripheral Atf3 + neuronal population is responsible for nerve regeneration at the early stage of nerve injury revealed by single-cell RNA sequencing : Peripheral Atf3 + neuronal population is responsible for nerve regeneration
Source: Acta Biochim Biophys Sin (Shanghai). 2024 Nov 13;57(3):424–36. doi: 10.3724/abbs.2024169 (PMC11986441; doi:10.3724/abbs.2024169)
Supplement: 24273Supplementary_figure_legends-20241029 [file 24273Supplementary_figure_legends-20241029.docx]

**
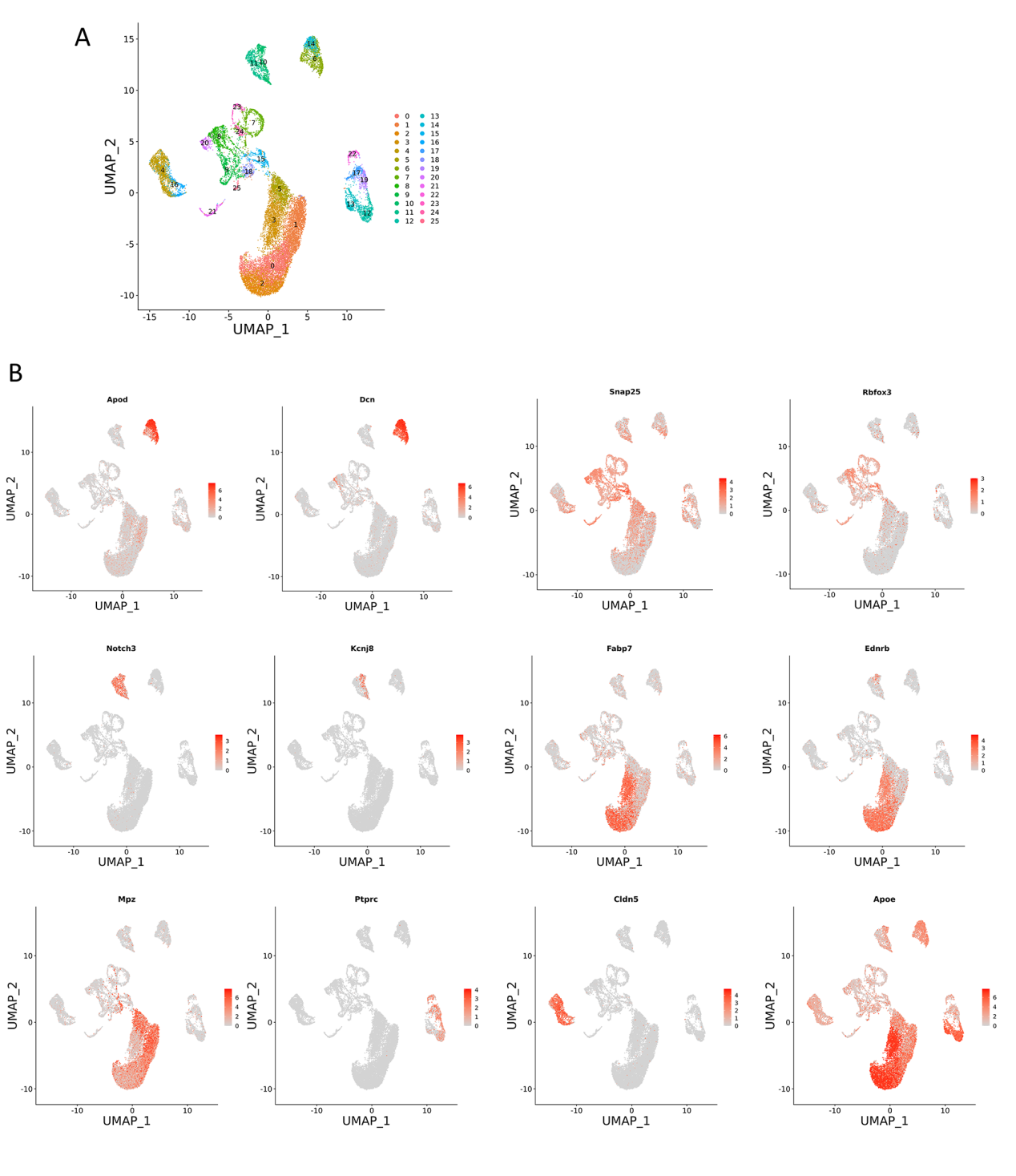
**

**Supplementary Figure S1. DRG cell types identification** (A) Preliminary DRG cell cluster results using a graph-based cluster method (resolution=0.8). (B) Feature heatmap showing the expression patterns of cell type-specific marker genes in all cell types. The color represents expression level.

**
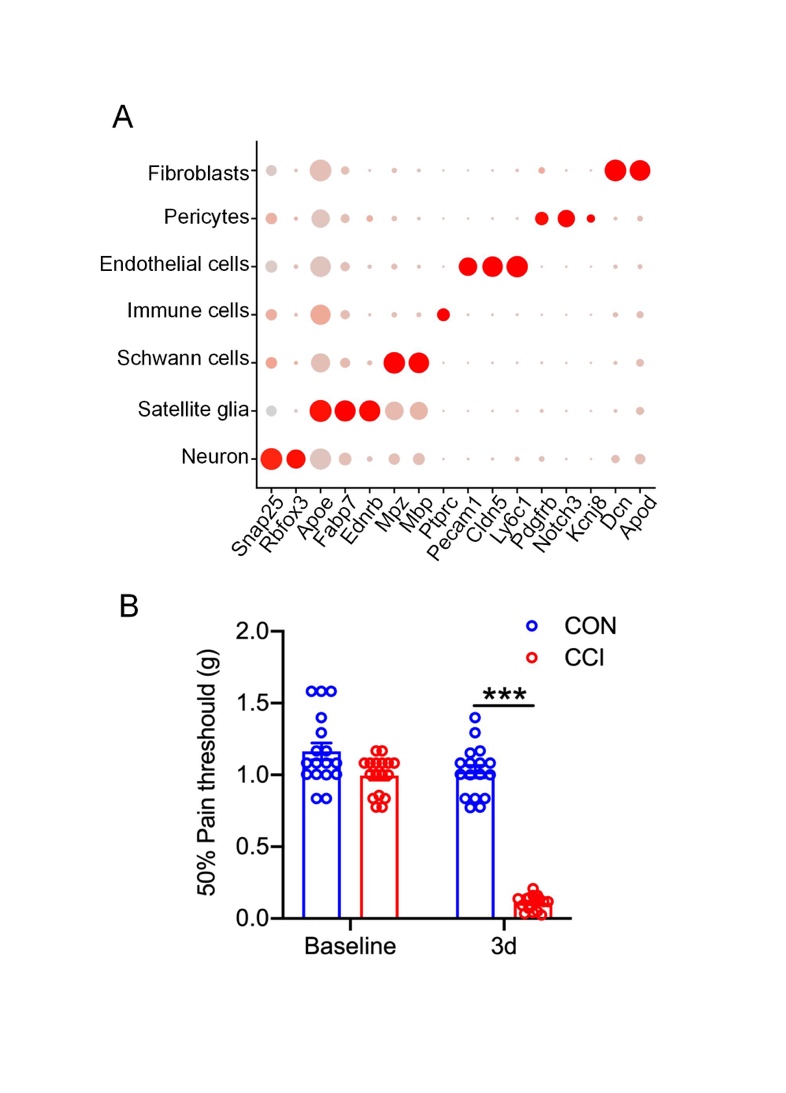
**

**Supplementary Figure S2. Decreased paw withdrawal threshold in CCI mice and the marker genes of DRG cells** (A) The marker genes of DRG cells. (B) Mechanical hypersensitivity 50% threshold of CON mice and day 3 post-CCI. 17 mice per group. ****P*<0.001; unpaired *t*-test. Data are presented as the mean ± SEM.

**
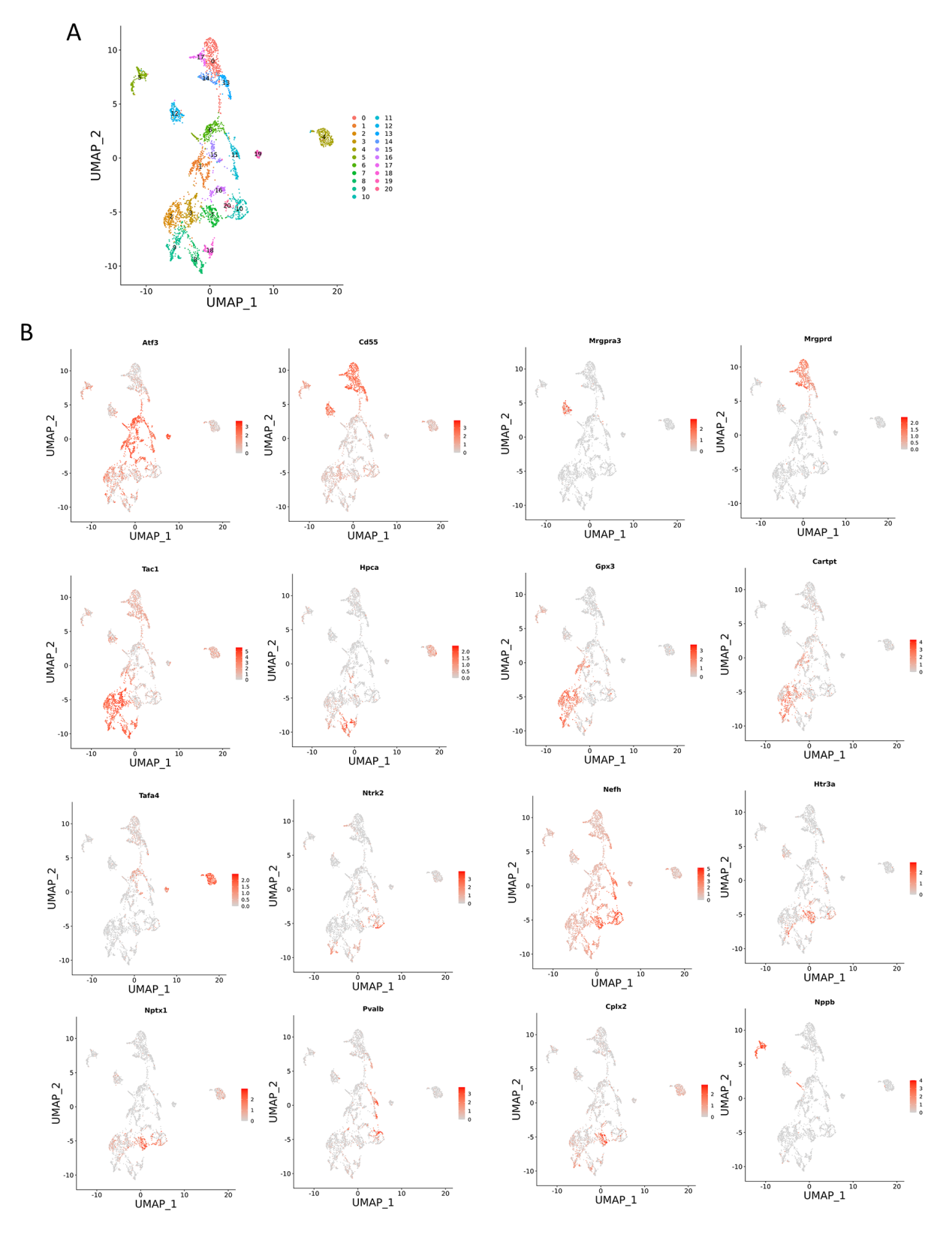
**

**Supplementary Figure S3. DRG neuronal subtypes identification** (A) Preliminary DRG neuronal subcluster results using a graph-based cluster method (resolution=0.8). (B) Feature heatmap showing the expression patterns of neuronal subtype-specific marker genes in all neuronal subtypes. The color represents expression level

**
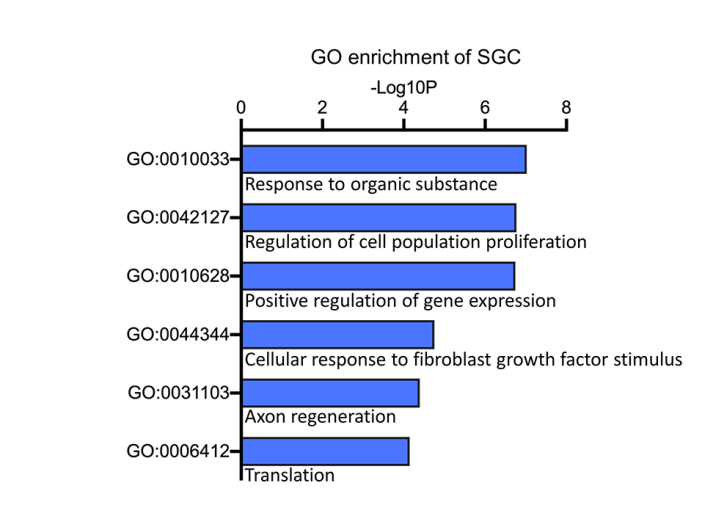
**

**Supplementary Figure S4. GO enrichment terms of BPs of DEGs in SGCs under CCI** Bar plot showing GO enrichment terms of BPs of DEGs in SGCs under CCI (*P*<0.001)
