# Supplementary material for: The peripheral Atf3 + neuronal population is responsible for nerve regeneration at the early stage of nerve injury revealed by single-cell RNA sequencing : Peripheral Atf3 + neuronal population is responsible for nerve regeneration
Source: Acta Biochim Biophys Sin (Shanghai). 2024 Nov 13;57(3):424–36. doi: 10.3724/abbs.2024169 (PMC11986441; doi:10.3724/abbs.2024169)
Supplement: Supplementary_Table_S1 [file Supplementary_Table_S1.docx]

**Supplementary Table S1. Sequences of primers used in this study**

| Gene | Primer sequence (5′→3′) |
| --- | --- |
| *Serpinb1a* | Forward: GACCAAACGTGAGAACTTGGA |
|  | Reverse: AGATCAGCCTTGCTACTGCTA |
| *Atf3* | Forward: TTTGCTAACCTGACACCCTTTG |
|  | Reverse: AGAGGACATCCGATGGCAGA |
| *Gal* | Forward: AGAGGCAGCGTTATCCTGCTA |
|  | Reverse: TCGCTAAATGATCTGTGGTTGTC |
| *Gadd45a* | Forward: AGACCGAAAGGATGGACACG |
|  | Reverse: GTACACGCCGACCGTAATG |
| *Sox11* | Forward: CGAGCCTGTACGACGAAGTG |
|  | Reverse: AAGCTCAGGTCGAACATGAGG |
| *Sprr1a* | Forward: TTGTGCCCCCAAAACCAAG |
|  | Reverse: GGCTCTGGTGCCTTAGGTTG |
| *Flrt3* | Forward: CCTCATCGGGACTAAAATTGGG |
|  | Reverse: CACAGCGACATACAGATGGAC |
| *Camk1* | Forward: AAGCAGGCGGAAGACATTAGG |
|  | Reverse: TCTGCCAGGATCACTTCTGAG |
| *Gap43* | Forward: AGATGGTGTCAAGCCGGAAG |
|  | Reverse: CGCCTTTGAGCTTTTTCCTTGT |
| *Tubb3* | Forward: CCCAGCGGCAACTATGTAGG |
|  | Reverse: CCAGACCGAACACTGTCCA |
| *S100a10* | Forward: GCTTACGTTTCACAGGTTTGC |
|  | Reverse: AAGCCCACTTTGCCATCTCG |
| *Kcnb2* | Forward: TGTGGACATTATCCGAAGCAAA |
|  | Reverse: TCTCGTGAGTGTTACAGTCGC |
| *Kcna2* | Forward: CACCCACAAGACACCTATGAC |
|  | Reverse: GTCTCTGGGAACTGGGCTAAG |
| *Kcnb1* | Forward: CTGTTGCATTGCCGGTGTC |
|  | Reverse: GCTCCTTGTAGAACTCGGAGAA |
| *Scn9a* | Forward: CCTTCCTCCGTGACCCTTG |
|  | Reverse: GTAGTCCAATTAGTGCGAACACA |
| *Scn10a* | Forward: TCCGTGGGAACTACCAACTTC |
|  | Reverse: TGCTTAGGTCTGCCCTTCTTG |
| *Scn8a* | Forward: ACCCGTACTATTTGACGCAGA |
|  | Reverse: TCCCTGTGAATGTGTACTCCA |
| *Gadd45g* | Forward: AAAGTCCTGAATGTGGACCCT |
|  | Reverse: AACGCCTGAATCAACGTGAAA |
| *Cacna2d1* | Forward: GCAGCCAACGGATTAAACCTG |
|  | Reverse: CGTGGGAATATGGACCGCT |
| *Cacnb4* | Forward: TTTGACGCCAAGGACTTTCTT |
|  | Reverse: GCCATGAAAACGGCCTCTT |
| *Csf1* | Forward: GTGTCAGAACACTGTAGCCAC |
|  | Reverse: TCAAAGGCAATCTGGCATGAAG |
| *Gpr151* | Forward: CGTGGGAAACCTGTGTGTGAT |
|  | Reverse: CAAACGCCCTTGGAGTATGC |
| *Gpr153* | Forward: CACACAGTTATGGGCATCTGG |
|  | Reverse: GGGTGTAGAAACGCTCGCTC |
| *Gpr158* | Forward: GACGTGGCCTCTTACCTCTAC |
|  | Reverse: GGCTAGGGACGGTGACTTTC |
| *Tnfrsf12a* | Forward: GTGTTGGGATTCGGCTTGGT |
|  | Reverse: GTCCATGCACTTGTCGAGGTC |
